# Supplementary material for: Dysfunction of ventrolateral striatal dopamine receptor type 2-expressing medium spiny neurons impairs instrumental motivation
Source: Nat Commun. 2017 Feb 1;8:14304. doi: 10.1038/ncomms14304 (PMC5296642; doi:10.1038/ncomms14304)
Supplement: Supplementary Information — Supplementary Figures and Supplementary Table. [file ncomms14304-s1.pdf]

Supplementary Figures

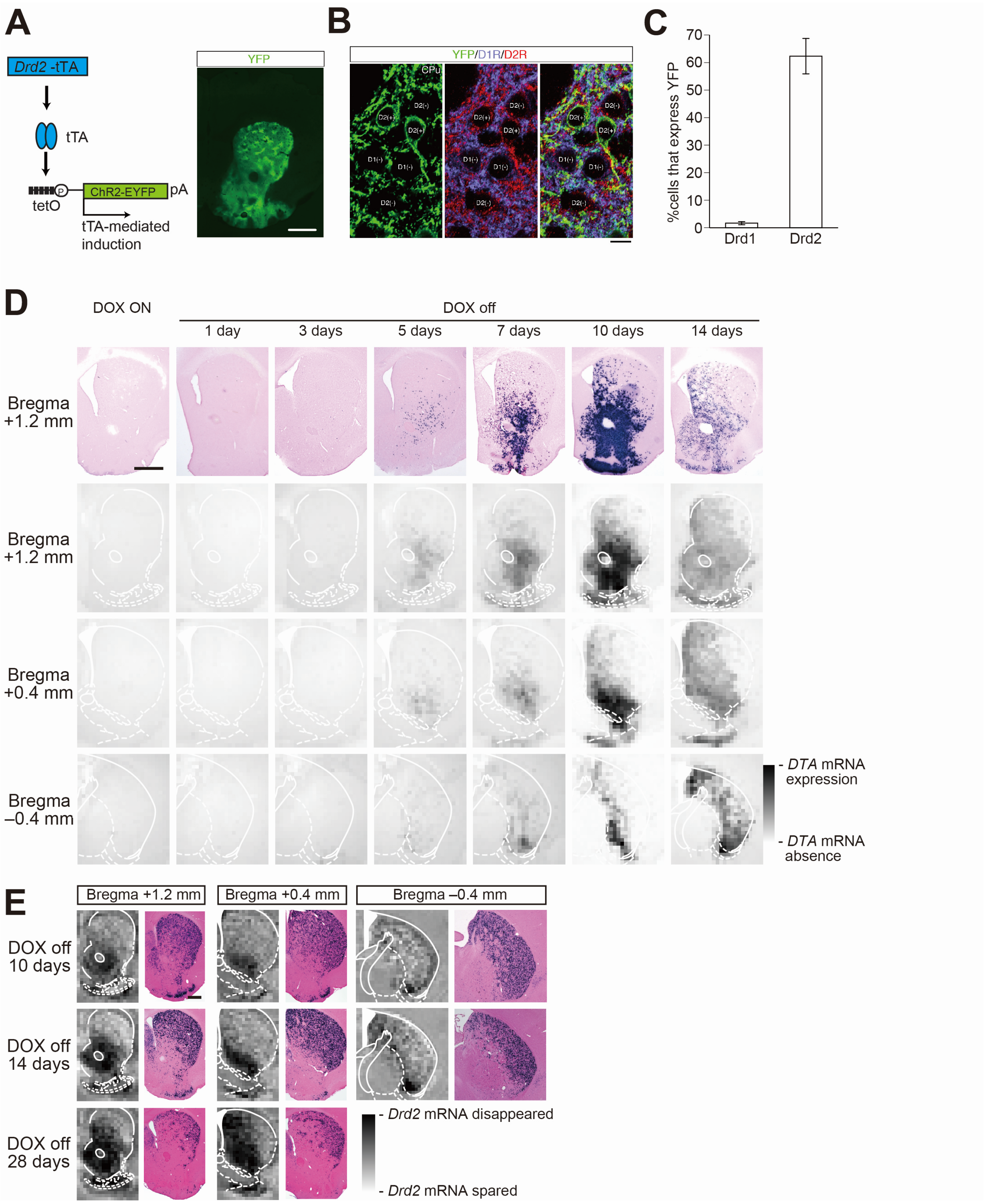

Supplementary Figure 1, Related to Figures 1 and 2

Characterization of *Drd2*-tTA BAC transgenic mice by crossing with KENGE-tet animal and pathological changes after doxycycline withdrawal in the striatum.

(**A**) tTA-mediated gene induction in combination with the highly efficient tetO line, KENGE-tetO-ChR2EYFP (Tanaka et al., 2012). In the bigenic *Drd2*-tTA::tetO-ChR2EYFP, EYFP immunoreactivity was seen across the whole striatal region.

(**B**) Triple labeling with EYFP, Drd1, and Drd2 in the striatum. Drd2-positive cells (red, D2-MSNs) expressed EYFP but D1-MSNs (blue) did not.

(**C**) Quantification. Error bars indicate S.E.M. (animal numbers= 5, 60-80 cells per each cell type per animal. t_8 = 9.399, $p < 0.001$). In terms of penetrance, 61% of Drd2-positive cells expressed EYFP. In terms of specificity, 97% of EYFP-positive cells are D2-MSNs.

(**D**) *DTA* mRNA expression after doxycycline withdrawal in the striatum. The top panels are representative images of *DTA* mRNA ISH. The lower gray-scale panels show averaged *DTA* mRNA expressing areas (n=4) at Bregma +1.2 mm, +0.4 mm, and -0.4 mm. The methods of determining averaged *DTA* mRNA expression were adapted from **"Determination of averaged *Drd2*-mRNA disappearance area"** in the **Methods**. Scale = 500 μm.

(**E**) *Drd2* mRNA disappearance after doxycycline withdrawal in the striatum. Each column contains images showing averaged *Drd2* mRNA disappearance area (n=5, left) and representative *Drd2* mRNA signal (right) at Bregma +1.2 mm, +0.4 mm, and -0.4 mm. *Drd2* mRNA disappearance area (black) increased after DOX-off treatment. Note that *Drd2* mRNA disappearance lay through the rostral to the caudal part of the VLS. See **Supplementary Figure 2** and **Methods** for the method of drawing. Scale = 500 μm.

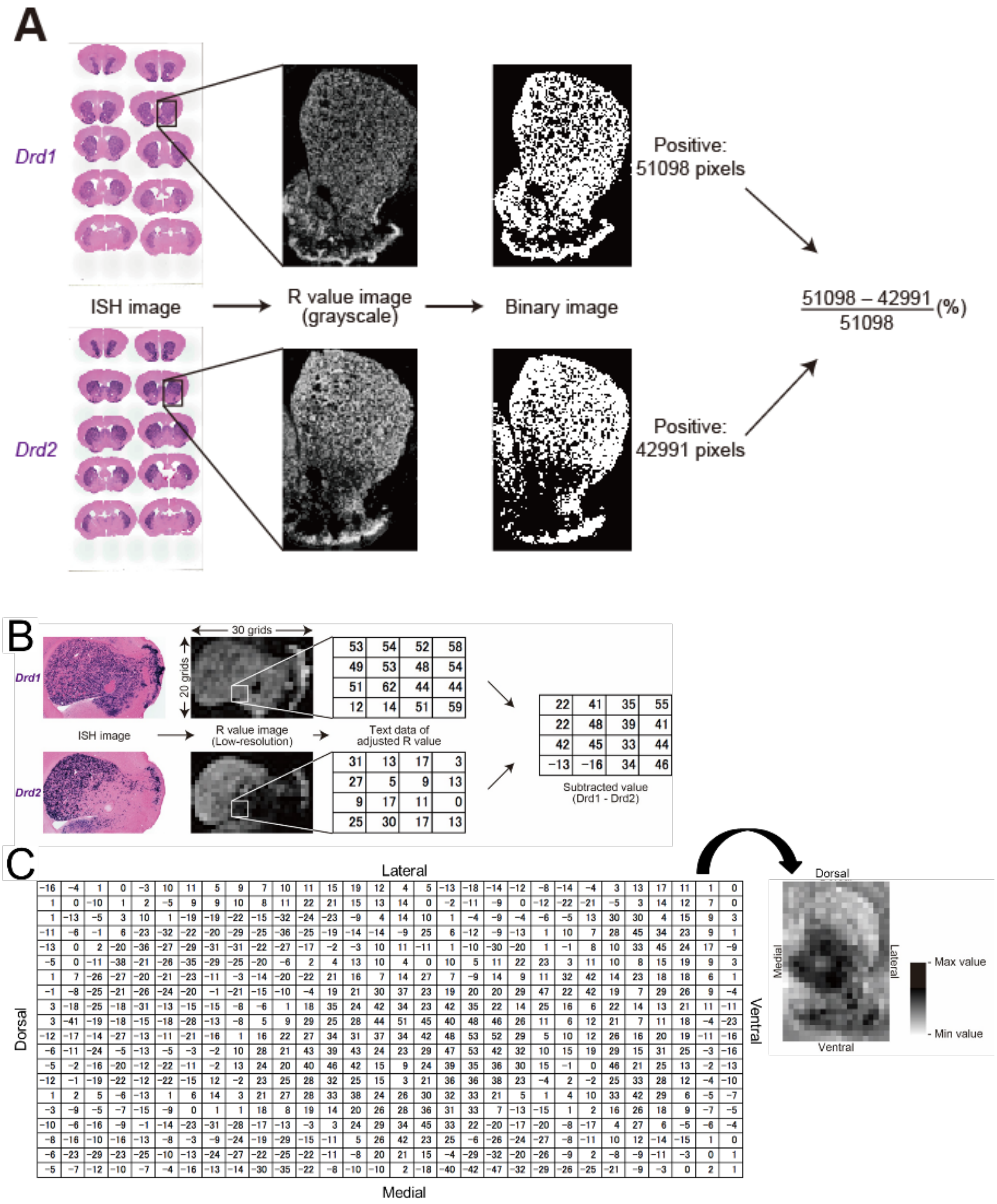

Supplementary Figure 2, Related to Figures 1 and 2

Workflow for the calculation of *Drd2* mRNA disappearance rate and quantification of *Drd2* mRNA disappearance

(**A**) Upper panel shows *Drd1* mRNA ISH image processing and lower panel shows that of

Drd2 mRNA. Colorimetric ISH images (left) were divided into RGB and R images were indicated in grayscale (middle). After image thresholding, binary images were obtained (right). Number of positive pixels in each slide was measured and *Drd2* mRNA disappearance value was calculated as (Drd1 pixel - Drd2 pixel) / Drd1 pixel.
(**B**) Quantification of *Drd2* mRNA disappearance. Original ISH images were divided into RGB and the resolution was reduced to 600 pixel/image ("R value image (Low-resolution)"). The value of each grid was imported to an Excel worksheet grid and was adjusted from 0 to 100 ("Text data of adjusted R value"). Large differences in Drd1 and Drd2 values indicated *Drd2* mRNA disappearance ("Subtracted value (Drd1 – Drd2)"). (**C**) Gray-scale image reconstruction. Averaged, normalized values are shown in the worksheet (20 x 30 grids). From the minimum value to the value 50, the linear grayscale was adapted and over 50 values showed black color. This image was used as **Figures 1C, 2A, and Supplementary Figure 1E**.

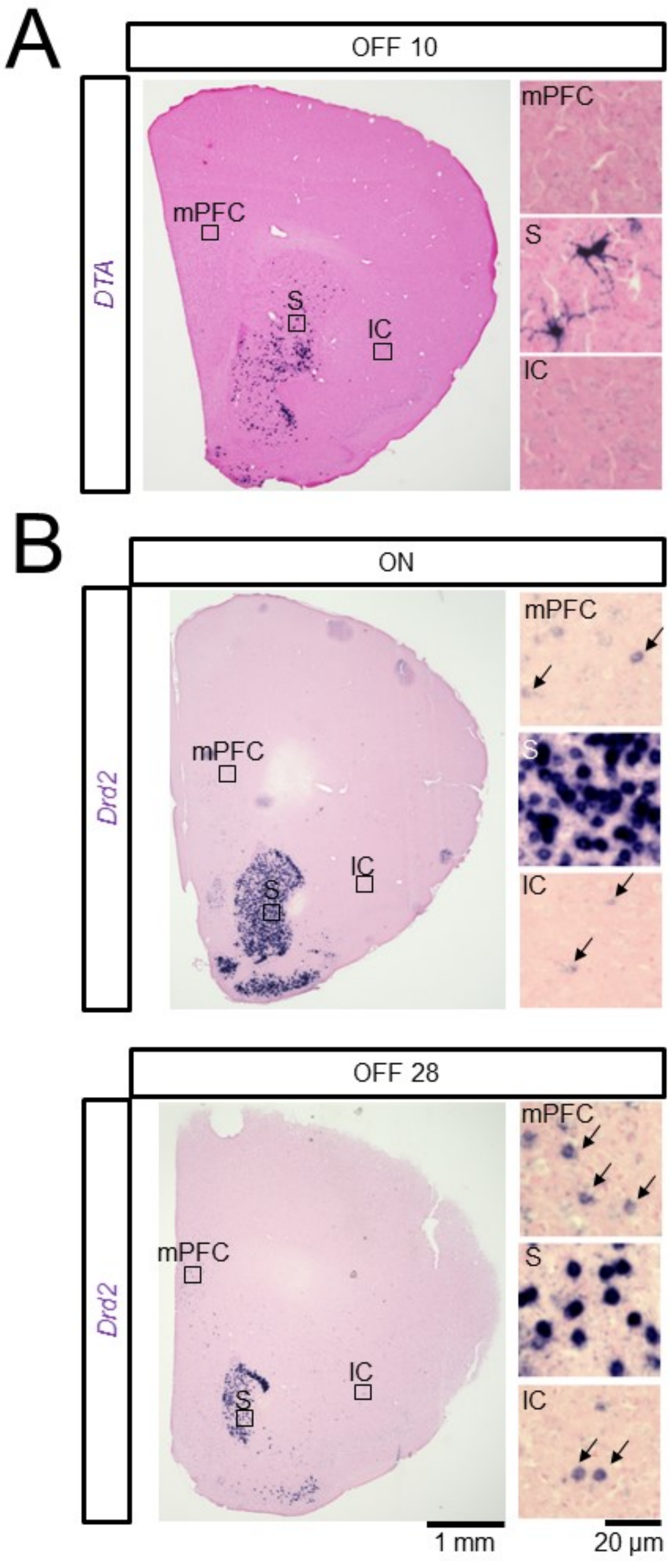

A
OFF 10
DTA
mPFC
S
IC
mPFC
S
IC
B
ON
Drd2
mPFC
S
IC
mPFC
S
IC
OFF 28
Drd2
mPFC
S
IC
mPFC
S
IC
1 mm
20 µm

Supplementary Figure 3, related to Figures 1

DTA induction is confined to the striatum

(**A**) No *DTA* mRNA induction in the mPFC or the insular cortex (IC) at DOX-off days 10 in D2-DTA.

(**B**) There are *Drd2* mRNA-positive cells in mPFC and IC (arrows), however, the numbers of *Drd2* mRNA-positive cells in both cortices did not change during DOX off regimen (quantification not shown). Upper: DOX on, lower: DOX off days 28. N=3 brains.

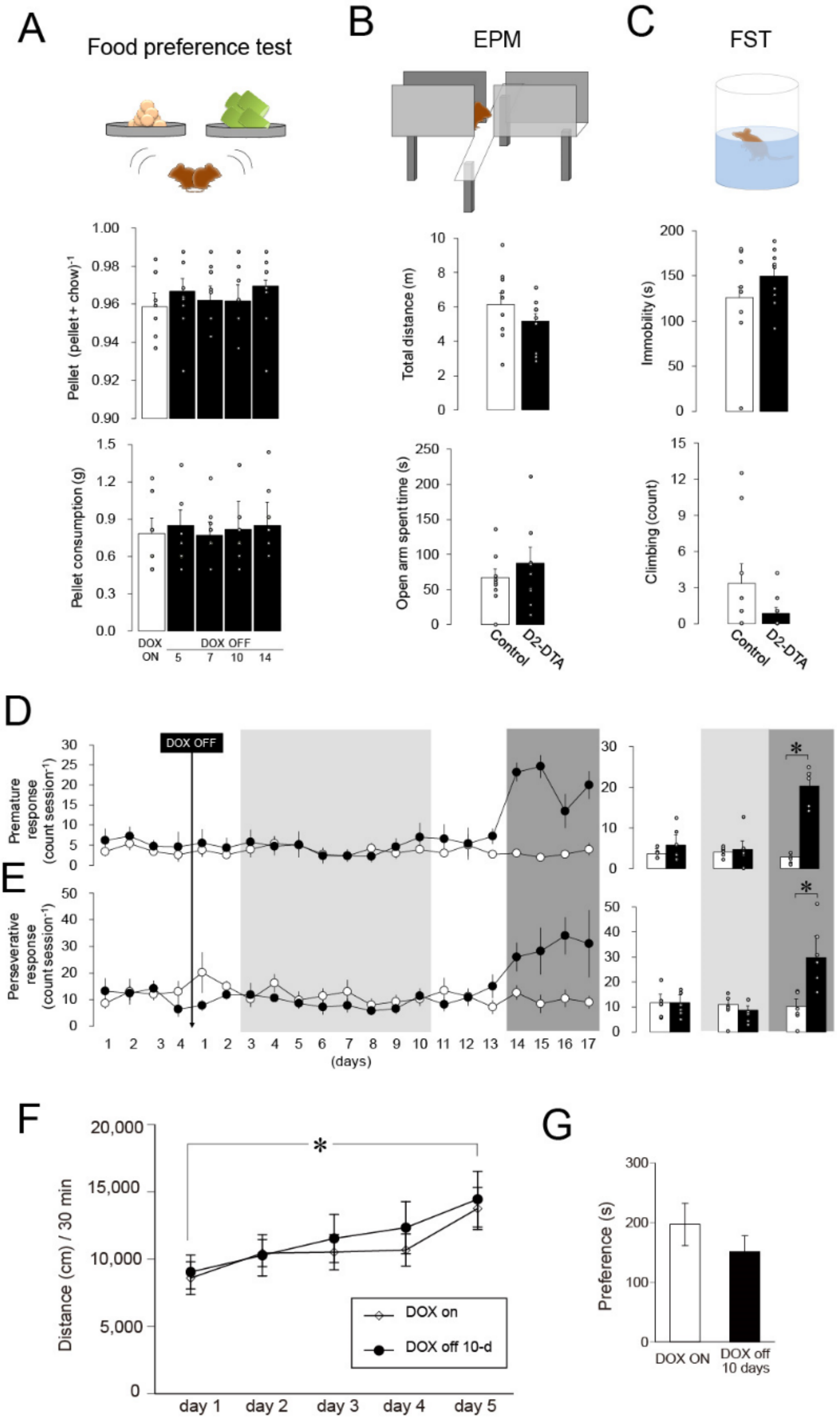
A
Food preference test
B
EPM
C
FST
Pellet (pellet + chow)-1
Pellet consumption (g)
DOX ON
DOX OFF
5 7 10 14
Total distance (m)
Open arm spent time (s)
Immobility (s)
Climbing (count)
Control
D2-DTA
D
E
DOX OFF
Premature response (count session-1)
Perseverative response (count session-1)
(days)
F
Distance (cm) / 30 min
day 1 day 2 day 3 day 4 day 5
DOX on
DOX off 10-d
G
Preference (s)
DOX ON
DOX off 10 days

Supplementary Figure 4, Related to Figure 4

Behavioral profile by loss-of-function in the VLS and/or VMS

(**A**-**C**) Loss-of-function of D2-MSNs in the VLS did not induce emotional dysregulation. Mice experiencing the DOX-off day 7 regimen displayed a comparable degree of anhedonia-like behavior (Palatable food preference, $F_{4, 40}$ = 0.759, P = 0.559; Palatable food consumption, $F_{4, 40}$ = 0.357, P = 0.837, *NS*, **A**) and a comparable degree of anxiety-like behavior in the elevated plus maze test (Total distance: t_{16} = 1.201, P = 0. 25, *NS*; Open arm spent time: t_{16} = 0.808, P = 0. 43, *NS*, **B**), and a comparable degree of despair-related behavior in the forced swim test (Immobility: t_{16} = 0.790, P = 0. 44, *NS*; Climbing: t_{16} = 1.542, P = 0. 14, *NS*, **C**).

(**D**-**E**) The temporal changes of behavioral parameters induced by DOX-off manipulations in the 3-CSRTT. The black circles represent data from the bigenic group (n=6) and the white circles represent data from monogenic controls (n=6). The bar graphs show the behavioral parameters averaged over -3 to 0 days before (phase **I**), 3 to 10 days after (phase **II**), and 14 to 17 days after (phase **III**) DOX termination in the 3-CSRTT. Increased premature responses (**D**) and perseverative responses (**E**) were observed only in phase **III**, when *Drd2* mRNA disappearance expanded into the VMS and DMS. Bars represent the mean and lines represent the SEM. *P < 0.05, with post hoc Student's t-test compared to the monogenic control.

(**F**-**G**) Ventrolateral D2-MSN ablation did not alter behavioral reactivity to methamphetamine (METH). Travel distance increases with repeated METH administration observed in DOX-off mice (10 days) was similar to that of controls (n=12 for DOX off group and n=11 for control, two-way repeated ANOVA revealed that there was no Day × Group interaction: $F_{1, 21}$=0.190, P=0.677; repeated ANOVA detected main effect of Drug for control group: $F_{1, 21}$=5.572, P<0.01; repeated ANOVA detected main effect of Drug for DOX off group: $F_{1, 21}$=6.976, P<0.01; multiple comparisons with Bonferroni method revealed a significant increase (P<0.05) of locomotor activity between Day 1 and Day 5 for both groups, **F**). DOX-off mice (10 days) showed similar preference for the METH-paired box as compared with controls (n=10 for each group, t_{18}= 1.034, p = 0.315, **G**). Error bars indicate S.E.M.

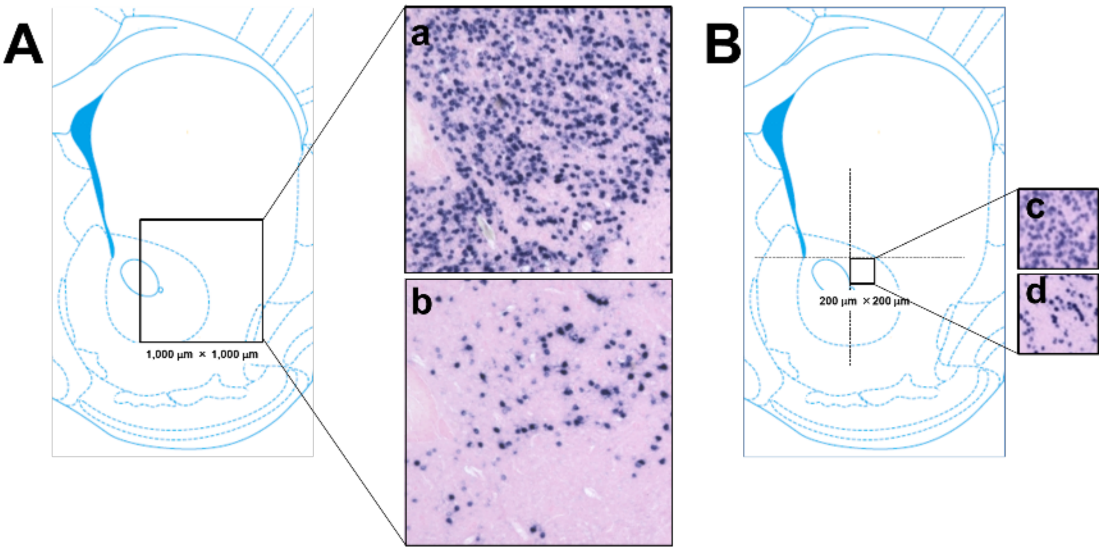

Supplementary Figure 5, Related to Figure 5

Areas for the quantification of mRNA expression

(**A**) Quantification of *Drd2* mRNA disappearance rate after 14 days of DOX restart. Mice trained on the PR task (n=6) were sacrificed after the final PR test and *Drd1* (**a**) and *Drd2* (**b**) mRNA images were obtained. *Drd2* mRNA disappearance rates in the black square were calculated in each animal (see **Methods, Quantification of *Drd2* mRNA disappearance rate after DOX withdrawal**).

(**B**) Quantification of *DTA* mRNA expression at 5 days off DOX. *Drd2* (**c**) and *DTA* (**d**) ISH images at 5 days off DOX (n=4) were used. ISH signals in the black square were analyzed. The *DTA* mRNA expression (%) was calculated as: [The number of *DTA* mRNA-positive neurons /the number of *Drd2* mRNA-positive neurons] × 100, and was averaged between animals. The number of *DTA* mRNA-positive cells was16.6% ± 5.7 of that of *Drd2*.

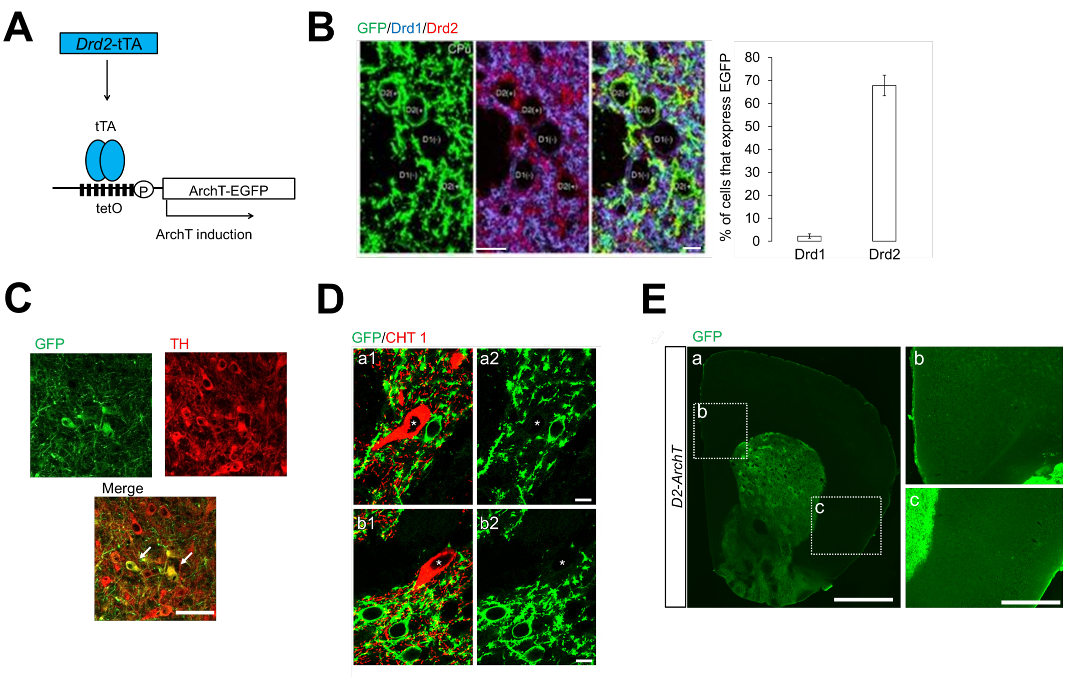

Supplementary Figure 6, related to Figure 6

Characterization of D2-ArchT mice.

(**A**) *Drd2*-tTA-dependent ArchT expression. (**B**) Triple labeling with GFP, Drd1, and Drd2 in the striatum. Drd2-positive cells (red, D2-MSNs) expressed GFP but D1-MSNs (blue) did not (animal numbers= 3, 40-50 cells per each cell type per animal. t_8 = 18.442, p < 0.001). Error bars indicate SEM. In terms of penetrance, 68% of Drd2-positive cells expressed EYFP. In terms of specificity, 97% of EGFP-positive cells are D2-MSNs. Scale bar = 1 mm and 10 μm (**C**) Double labeling with GFP and TH in the VTA of D2-ArchT mouse. 90% of GFP-positive cells (n= 7.0 ± 2.3) were labeled with TH, and 7% of TH-positive cells (n=76.8 ± 8.6) were labeled with GFP (3 animals). Scale bar = 20 μm.(**D**) Double labeling with GFP and CHT 1 (a1, a2, b1, and b2) in the striatum of D2-ArchT mouse. Cholinergic interneurons did not express GFP. Scale bar = 10 μm. (**E**) No GFP expression was detected in cortical neurons in mPFC (a) or IC (b). These data indicated that the illumination did not affect the activity of cortical neuron terminals in the striatum. Scale bar = 500 μm.

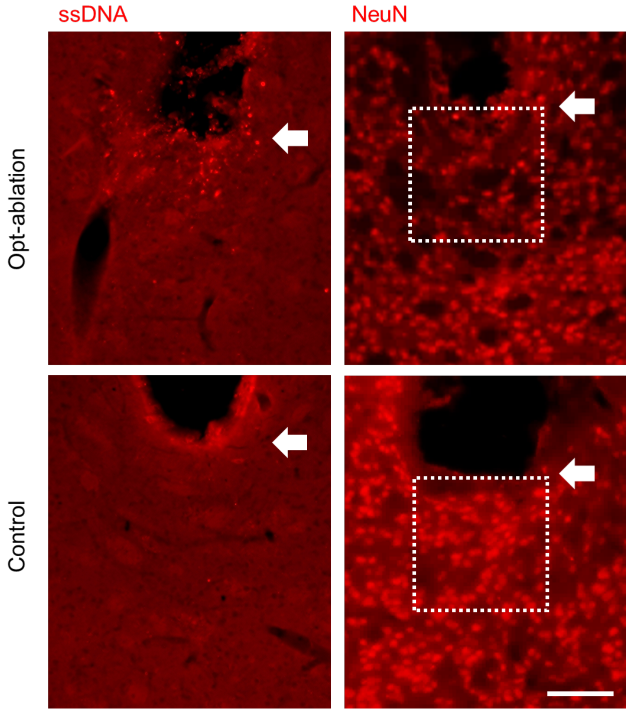

Supplementary Figure 7, Related to Figure 6

Optogenetic ablation in D2-ArchT mice.

The 3-hr yellow light (upper panels) illumination induced cell death, which was supported with the expression of single strand DNA (ssDNA) and a decreased number of NeuN positive cells (Control: 62.8 ± 3.6, Opt-ablation: 29.3 ± 3.8, mean ± S.E.M., cells/white dash square). White dash square = 400 μm × 400 μm. White arrows indicate the tip of optic fibers. Scale=20 μm

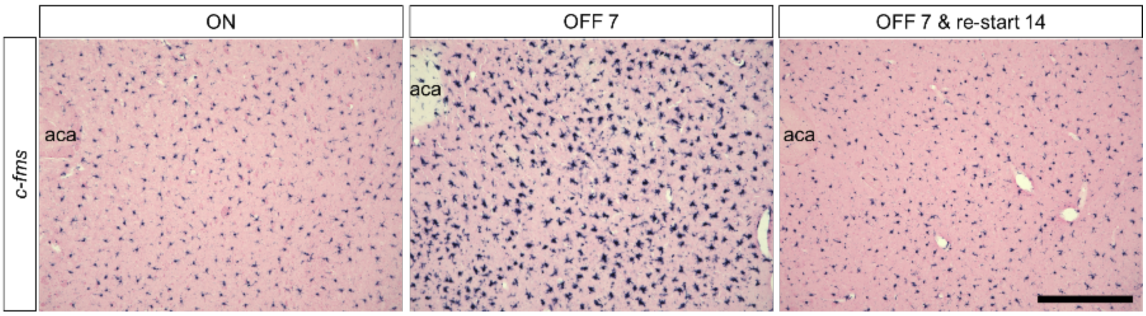

Supplementary Figure 8, Related to the 6th paragraph of Discussion
Alleviation of microglial activation after DOX-restart.

In situ hybridization for *c-fms* (the marker of microglia) shows resting microglia (DOX-on, left), activated microglia (DOX-off day 7, middle), and resting-like microglia (DOX-off day 7 and restart day 14, right) in the VLS of D2-DTA mice. Scale bar = 500 μm

Supplementary Table

Supplementary table 1

Specificity of *tTA* expression and penetrance of tTA-mediated gene induction

			tTA expression	*DTA* induction	ArchT-EGFP induction
Striatum	*Drd2*-positive cells	D2-MSNs	+	+ (Fig 1B)	+ (Fig S5B)
		Cholinergic interneurons	- (Fig 1H)	- (Fig 1I)	- (Fig S5D)
	Drd2-negative cells	D1-MSNs	-	- (Fig 1K)	- (Fig S5B)
Outside striatum	*Drd2*-positive cells	DA neurons	+	- (Fig 1J)	+/- (Fig S5C)
		IC neurons	-	- (Fig S1F)	- (Fig S5E)
		mPFC neurons	-	- (Fig S1F)	- (Fig S5E)
		Mossy cells	-	-	-

Note: +: detected, -: not detected, +/-: detected but unlikely functional, parenthesis shows related data.
